# Gibberellins and heterosis of plant height in wheat (*Triticum aestivum *L.)

**DOI:** 10.1186/1471-2156-8-40

**Published:** 2007-06-29

**Authors:** Yi Zhang, Zhongfu Ni, Yingyin Yao, Xiuling Nie, Qixin Sun

**Affiliations:** 1 Department of Plant Genetics & Breeding and State Key Laboratory for Agrobiotechnology, China Agricultural University, Beijing 100094, China; 2 Key Laboratory of Crop Heterosis and Utilization (MOE), Key Laboratory of Crop Genomics and Genetic Improvement (MOA) and Beijing Key Laboratory of Crop Genetic Improvement, Beijing, China

## Abstract

**Background:**

Heterosis in internode elongation and plant height are commonly observed in hybrid plants, and higher GAs contents were found to be correlated with the heterosis in plant height. However, the molecular basis for the increased internode elongation in hybrids is unknown.

**Results:**

In this study, heterosis in plant height was determined in two wheat hybrids, and it was found that the increased elongation of the uppermost internode contributed mostly to the heterosis in plant height. Higher GA_4 _level was also observed in a wheat hybrid. By using the uppermost internode tissues of wheat, we examined expression patterns of genes participating in both GA biosynthesis and GA response pathways between a hybrid and its parental inbreds. Our results indicated that among the 18 genes analyzed, genes encoding enzymes that promote synthesis of bioactive GAs, and genes that act as positive components in the GA response pathways were up-regulated in hybrid, whereas genes encoding enzymes that deactivate bioactive GAs, and genes that act as negative components of GA response pathways were down-regulated in hybrid. Moreover, the putative wheat GA receptor gene *TaGID1*, and two GA responsive genes participating in internode elongation, *GIP *and *XET*, were also up-regulated in hybrid. A model for GA and heterosis in wheat plant height was proposed.

**Conclusion:**

Our results provided molecular evidences not only for the higher GA levels and more active GA biosynthesis in hybrid, but also for the heterosis in plant height of wheat and possibly other cereal crops.

## Background

Heterosis or hybrid vigour was defined as the better performance of hybrid plants over its parental inbreds in terms of viability, growth and productivity. Hybrid cultivars have been used in many crop plants and have made significant contribution to the world food supply [1]. However, molecular basis of heterosis is still poorly understood. Recent studies suggested that differential or nonadditive gene expression in hybrid might contribute to the heterosis [2-6]. By using diallel crosses, it was shown that some of the differential expression patterns detected between hybrids and their parents in leaf tissues were significantly correlated with heterosis observed in agronomic traits in rice [7] and wheat [3]. Subsequent identification of the differentially expressed genes indicated that diverse categories of genes are involved [4-6], [8-10]. Since all the genes in hybrids are derived from its parental inbreds, the phenotypic differences between hybrids and their parents, or heterosis, could be best explained by the spatio-temporal differences in gene expression [3].

Plant height is one of the typical traits showing heterosis in many crop plants. In maize and sorghum, the mid-parent heterosis in plant height can be 40% and 16%, respectively [11]. In wheat, over 10% of mid-parent heterosis was observed in different hybrids [12]. The heterosis in plant height mainly results from increased internode elongation other than increases in the number of internodes. Therefore, investigation on the mechanisms of increased internode elongation in hybrids will certainly facilitate our understanding of molecular basis of heterosis in plant height.

Gibberellins (GAs) are plant hormones that participate in regulation of many growth and developmental processes in plants [13,14], and are especially important in regulating stem elongation [15-21]. Therefore, it is reasonable to look for the relationship between GAs and heterosis in plant height. Previous studies indicated that GA levels are correlated with the vigorous plant growth observed in hybrid F_1 _plants [11,22-24]. Three lines of evidences are available to support this relationship. Firstly, hybrids have higher GA levels than parental inbreds in maize [11,24,25], sorghum [26], poplar [27], black spruce [28] and interspecific hybrid between *Liriodendron chinense *and *L. tulipifera *[29]. The higher GA levels are correlated with faster shoot growth rate in hybrids [11]. Secondly, maize inbreds are more responsive than their hybrids to the application of exogenous GA [11,25], suggesting that the growth of maize inbreds is limited by a deficiency of endogenous GAs, whereas the hybrids possess GA at near saturation [24]. And finally, the conversion of GA20, the precursor of bioactive GAs, to GA1, the bioactive GA, is more rapid in the shoot cylinders of hybrid than in the shoot cylinders from inbreds [30,31]. Collectively, these studies suggested that GA content and metabolism are positively correlated with faster shoot growth rate of hybrid, or heterosis. However, to our best knowledge, there has been no study on expression patterns of genes related to GA metabolism between hybrids and their parental inbreds.

The GA biosynthesis pathway has been extensively studied, and most of the genes encoding enzymes in each step of GA biosynthesis and catabolism pathways have been identified in the model plant species Arabidopsis and rice [13,14,18,32]. Recently, GIBBERELLIN INSENSITIVE DWARF1 has been identified as a soluble GA receptor in rice [33]. We hypothesized that higher GA contents in hybrid plants could result from the differential expression of genes that participate in the regulation of GA biosynthesis and catabolism, and increased shoot or stem growth rate of hybrids could be related to the differential expression of genes participating in regulation of GA response pathways. In order to provide evidences, in this study, by using the uppermost internode tissues of wheat, we examined expression patterns of genes participating in both GA biosynthesis and GA response pathways between a wheat hybrid and its parental inbreds. Our results indicated that among the 18 genes analyzed, genes encoding enzymes that promote synthesis of bioactive GAs, and genes that act as positive components in GA response pathways were up-regulated in hybrid, whereas genes encoding enzymes that deactivate bioactive GAs, and genes that act as negative components of GA response pathways were down-regulated in hybrid. Taken together, these data provided molecular evidences not only for the higher GA levels and more active GA biosynthesis in hybrid, but also for the heterosis in plant height of wheat and possibly other cereal crops.

## Results

### Heterosis in plant height and internode length

The plant height and length of internodes in two wheat hybrids and their parental inbreds were measured. Significant heterosis in plant height was observed (Tables 1 and 2). The HP (high parent) heterosis in plant height for hybrid 309-1/AIM-11 was 18.36% in greenhouse condition, whereas, the HP heterosis for hybrid Ai9/Jiai8 were 22.42% and 18.84% in the greenhouse and field growing conditions, respectively. The lengths of five internodes were also determined for each genotype, and it was found that four of the five internodes in length showed significant HP heterosis. For both hybrids, the uppermost internode or the first internode showed the largest heterosis, followed by second and third internodes (Tables 1 and 2), suggesting that the increased elongation of the uppermost internode is the major contributor to the heterosis in plant height. However, the length of the fifth internode showed no significant heterosis (Tables 1 and 2).

**Table 1:** Heterosis in plant height and internode length in hybrid 309-1/AIM-11

	Female parent(309-1)	hybrid	male parent(AIM-11)	HP heterosis
ear	5.34 ± 0.31	5.76 ± 0.25*	5.2 ± 0.25	7.86%
1th internode	14.98 ± 1.94	20.78 ± 4.42**	14.45 ± 1.33	38.72%
2th internode	13.23 ± 1.7	14.85 ± 0.94**	13.07 ± 1.0	12.24%
3th internode	8.98 ± 0.56	9.98 ± 0.62**	8.33 ± 0.3	11.36%
4th internode	6.43 ± 0.15	6.87 ± 0.1**	6.15 ± 0.3	6.84%
5th internode	2.23 ± 0.12	2.35 ± 0.1	2.25 ± 0.11	4.44%
plant height	51.19 ± 7.57	60.59 ± 11.3**	49.25 ± 9.39	18.36%

*, ** : different between hybrid and high parent at p < 0.05 and p < 0.01, respectively

**Table 2:** Heterosis in plant height and internode length in hybrid Ai9/Jiai8

	female parent (Ai9)	hybrid	male parent (Jiai8)	HP heterosis
ear	5.36 ± 0.19^a)^	5.76 ± 0.18	5.5 ± 0.18	4.73%
	8.66 ± 0.21^b)^	10.30 ± 0.23	9.88 ± 0.20	4.25%
1th internode	13.92 ± 1.21	22.23 ± 2.35**	16.36 ± 1.82	35.88%
	18.15 ± 1.68	27.68 ± 2.25**	20.35 ± 2.13	36.02%
2th internode	12.84 ± 1.13	16.25 ± 1.85**	13.72 ± 1.24	18.44%
	16.56 ± 1.23	21.78 ± 1.96	18.23 ± 1.37	19.47%
3th internode	8.32 ± 0.52	9.62 ± 0.86**	8.50 ± 0.93	13.18%
	10.26 ± 0.98	12.33 ± 1.23	11.18 ± 1.12	10.29%
4th internode	6.30 ± 0.28	7.33 ± 0.68**	5.60 ± 0.27	16.35%
	8.66 ± 0.63	9.78 ± 0.76	9.03 ± 0.57	8.31%
5th internode	2.20 ± 0.28	2.65 ± 0.19	2.44 ± 0.28	8.61%
	3.06 ± 0.32	3.67 ± 0.27	3.31 ± 0.33	10.88%
plant height	48.94 ± 8.41	63.84 ± 5.5**	52.15 ± 7.81	22.42%
	**65.35 **± 6.22	85.54 ± 7.35**	71.98 ± 7.18	18.84%

*, ** : different between hybrid and high parent at p < 0.05 and p < 0.01), respectively.

a) Data from greenhouse experiment in 2005; b) Data from field experiment in 2006/07

### Contents of GA3 and GA4 in hybrid Ai9/Jiai8 and its parents

Previous studies in maize and sorghum indicated that hybrid contained higher endogenous GAs level than its parents, which was correlated with heterosis in shoot cylinder height [25,26]. Since GAs play important roles in regulating stem elongation, and the length of uppermost internode in wheat hybrids showed the most significant heterosis, we determined the concentrations of two bioactive GAs, GA_3 _and GA_4_, in the uppermost internode tissues in a wheat hybrid Ai9/Jiai8 and its parents using GC-MS-SIM. The basal 1 cm portion of the uppermost internode contains IM and elongation zone and is responsible for stem elongation [34], and therefore used for GA content analysis. We found that hybrid had significantly higher GA_4 _content than its parents, with the HP heterosis of 43.80% (Table 3). However, heterosis in GA_3 _content was not statistically significant (Table 3). These results confirmed previous findings that hybrids contained higher level of bioactive GAs as compared to their parents.

**Table 3:** GA_3 _and GA_4 _contents (ng/g FW) in IM and elongation zone

	Ai9	Ai9 × Jiai8	Jiai8	HP heterosis
GA_3_	1.404 ± 0.047	1.528 ± 0.114	1.462 ± 0.034	4.51%
GA_4_	0.452 ± 0.072	0.650 ± 0.057*	0.408 ± 0.024	43.80%

*, different between hybrid and high parent at p < 0.05

### Differential expression of genes in GA biosynthesis between hybrid Ai9/Jiai8 and its parents

Previous studies suggested that the higher level of GAs content in hybrid was associated with faster conversion of the precursor of bioactive GAs to the bioactive forms. We further hypothesized that genes in GAs biosynthesis might be differentially expressed, which, in turn, could result in the differences in GAs content between hybrid and parents.

Up to date, most of the genes encoding enzymes of GA biosynthesis have been identified from wheat and other plant species, which makes it possible to determine the expression patterns of these genes between hybrid and parents. The biosynthesis of GA in higher plants can be divided into three stages. CPS, KS, KO and KAO are involved in the first and second stages, whereas GA20ox, GA3ox and GA2ox are involved in the third stage of GA biosynthesis. In this study, we detected expression patterns of genes encoding KS, KAO, GA20ox, GA3ox, GA2ox, RSG (the positive regulator of *KAO*) and 14-3-3 (the negative regulator of *RSG*) between a hybrid and its parents (Table 4).

**Table 4:** Differential expression of target gene relative to β-actin between hybrid and parents.

Genes	Ai9 (female)	hybrid	Jiai8(male)
*RSG*	0.155 ± 0.014	0.122 ± 0.016	0.0772 ± 0.0087
*14-3-3*	1.119 ± 0.11	1.096 ± 0.17	1.514 ± 0.067*
*KS*	5.31E-05 ± 4.48E-06	4.78E-05 ± 5.48E-06	8.67E-05 ± 3.12E-06**
*KAO*	1.08E-05 ± 1.47E-07**	3.52E-05 ± 5.24E-06	2.00E-05 ± 3.6E-06*
*TaGA20ox-2*	0.000797 ± 0.0002**	0.00647 ± 0.0013	0.00497 ± 0.00012
*TaGA20ox1D*	0.00264 ± 0.00058*	0.00605 ± 0.0016	0.00347 ± 0.00066*
*TaGA3ox2-1*	0.0021 ± 0.0006*	0.00408 ± 0.0007	0.00077 ± 7.4E-05**
*TaGA3ox2-2*	0.0067 ± 0.00049**	0.011 ± 0.00037	0.0031 ± 0.0002**
*TaGA3ox2-2*	0.00092 ± 9.2E-05**	0.0019 ± 0.00023	0.00096 ± 0.00022**
*TaGA2ox-1*	0.0019 ± 0.00036	0.0017 ± 8.1E-05	0.0022 ± 8.6E-05**
*GAI*	0.270 ± 0.031	0.217 ± 0.037	0.0808 ± 0.0075**
*XET*	0.324 ± 0.022**	0.684 ± 0.069	1.00 ± 0.24
*GAMYB*	0.00045 ± 6.4E-05**	0.0019 ± 0.00025	0.00072 ± 6.4E-05**
*GIP*	0.045 ± 0.002**	0.108 ± 0.007	0.093 ± 0.007*
*TaGID1*	0.055 ± 0.0027*	0.11 ± 0.029	0.029 ± 0.0029**

Average of the three 2 ^-ΔCT ^values for each sample was listed. t-test was used for statistical analysis.

*, ** different between hybrid and parent at p < 0.5 and p < 0.01, respectively.

Real time quantitative PCR analysis indicated that *KS *gene was down-regulated in wheat hybrid as compared to its parents, but the difference between hybrid and the lower parent was not significant. *KAO *gene was significantly up-regulated in hybrid. Three wheat homoeologues of *TaGA20ox1 *had been cloned in wheat and were mapped on chromosome 5BL, 5DL and 4AL, respectively [35]. We found that expression of *TaGA20ox1D *was significantly up-regulated in hybrid, whereas expression of *TaGA20ox1A *and *TaGA20ox1B *were not detected. The expression of putative wheat *TaGA20ox2 *gene was also up-regulated in wheat hybrid, though difference between hybrid and the higher parent was not significant. Three homoeologues of *TaGA3ox2 *had also been cloned [35]. Real-time quantitative PCR analysis indicated that all the 3 homoeologues of *TaGA3ox2 *were expressed in wheat internode tissues and were significantly up-regulated in hybrid (Table 4). Analysis also showed that the putative *TaGA2ox-1 *was slightly down-regulated in hybrid. *RSG*, the positive regulator of *KAO*, was expressed as mid-parent value in hybrid, and the difference between hybrid and parents was not significant. *14-3-3*, the negative regulator of *RSG*, was down-regulated in hybrid (Table 4).

### Differential expression of genes in GA response pathway between hybrid Ai9/Jiai8 and its parents

Studies revealed that GA response pathways were linked tightly to its biosynthesis and catabolism, and played crucial roles in GA-regulated developmental processes [14]. Different components of GA response pathway and the down-steam target genes had been identified [14,36-38]. To further determine whether genes in GA response pathways are differentially expressed and to elucidate their possible roles in heterosis of plant height, the expression patterns of several GA response pathway genes were determined, including the soluble GAs receptor *GID1*, the negative regulator *GAI*, the positive regulator *GAMYB*, and GA-regulated target genes such as expansin, endoxyloglucan transferase (XET) and gibberellins induced protein (*GIP*). Analysis indicated that both the putative wheat homologs of *GID1 *and *GAMYB *were up-regulated in hybrid, whereas *GAI *(Rht-1) was expressed as mid-parent value, the putative wheat homolog of *GIP *was up-regulated in hybrid, *XET *was expressed at the level close to the higher parent (Table 4). In our previous study, semi-quantitative PCR revealed that four members of β-expansins were also up-regulated in hybrid [39].

### Response of GA response pathway genes to application of exogenous GA

To verify whether the genes we detected in GA response pathway were responsive to GA, GA_3 _was applied to the internode sections and response to exogenous GA application was determined. Real-time quantitative PCR analysis indicated that transcript level of *GAI *was decreased to about half of control within 6h of GA_3 _treatment, and transcript level of *GAMYB *and *GIP *was significantly increased after GA_3 _treatment, whereas transcript level of *XET *was slightly down-regulated, but this decrease was not significant (Table 5).

**Table 5:** Expression of GAs responsive genes with the application of exogenous GA_3_

Gene	control	GA_3 _treatment
*GAI*	0.37 ± 0.019	0.18 ± 0.016 **
*Myb*	0.0017 ± 0.00019	0.0023 ± 0.00019 *
*XET*	0.09 ± 0.014	0.097 ± 0.017
*GIP*	0.38 ± 0.027	0.53 ± 0.055 *

Average of the three 2 ^-ΔCT ^values from 3 repeated PCR were listed. t-test was used for statistical analysis.

*, ** different between hybrid and parent at p < 0.5 and p < 0.01, respectively.

### Anatomy of internode in hybrid Ai9/Jiai8 and its parents

**Figure 1:**
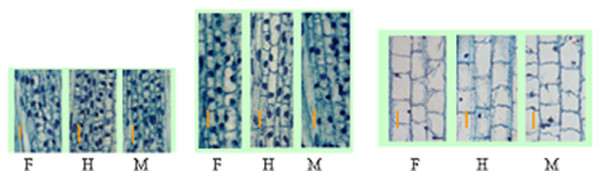
Sections of the uppermost internodes of hybrid Ai9/Jiai8 and its parents. F for the female parent, H for the hybrid, and M for the male parent. (A) Longitudinal sections through the IM. Ba = 0 μm. (B) Longitudinal sections through the elongation zone. Bar = 20 μm. (C) Longitudinal sections through the differential zone. Bar = 45 μm.

GAs promotes stem elongation by enhancing cell division and elongation. Since internode from hybrid contains higher level of bioactive GAs, and in this study we found that most of the positive components of GA response pathways were up-regulated in hybrid, we speculated that the increased internode elongation observed in hybrid could result from the enhanced cell division and cell elongation. To provide evidence, internode sections of 1cm above the uppermost node, which contains IM and elongation zone, were sectioned, and cell number and length were determined. Internode sections from differentiation zone were also observed. It was found that cell number in the IM zone of hybrid was 73 within 500 μM × 80 μM scope, whereas this number was 64 and 74 for the two parents, respectively (Fig. 1). The average cell length in elongation zone was 50 μm in hybrid, but was 50 μm and 45.45 μm for the two parents, respectively (Fig. 1). In our observation, the average cell length in differentiation zone was 89 μm in hybrid, and but was 73 μm and 71 μm for the two parents, respectively (Fig. 1), with the HP heterosis of 21.9%.

## Discussion

### Higher GAs contents in hybrid might result from differential expression of genes involved in GA biosynthesis

Previous studies in maize, sorghum, poplar, black spruce and *Liriodendron *indicated that endogenous GAs content in hybrid was usually higher than their parental inbreds [24-29], and the higher GA levels were correlated with faster shoot and stem growth rate in hybrid [11,22]. Further studies showed that the conversion of GA_20_, the precursor of bioactive GAs, to GA_1_, bioactive GA, was more rapid in the shoot cylinders of hybrid than in the shoot cylinders from inbreds [30,31], which provided physiological and biochemical basis of higher GAs contents observed in hybrids. In this study, higher GAs contents were also observed in the internode tissues of a wheat hybrid. In crops such as rice and wheat, stem elongation is caused by mitotic activity of the cells in the IM and by the elongation of these cells in the elongation zone immediately above the IM [34]. Endogenous GA in this region directly promotes internode elongation. Thus, the higher endogenous GA level in IM and elongation zone of wheat hybrid might promote its cells to divide and elongate more vigorously, and generate more and/or longer cells in comparison with parents. Histological observation in this study provided further evidence that increased GA concentration could enhance cell division and cell elongation in wheat hybrid.

However, molecular basis of higher GAs contents and faster conversion to bioactive forms of GAs in hybrids are still unknown. Intensive investigations in model plant species, such as Arabidopsis and rice, have identified most of the genes encoding enzymes in each steps of GA biosynthesis [13,14,18,32], and manipulation of some of these genes caused changes in GA contents as well as morphological changes, including changes in plant height. The biosynthesis of GA in higher plants can be divided into three stages. In the first stage, geranylgeranyl diphosphate is converted to *ent*-kaurene by CPS and KS, in the second stage, *ent*-kaurene is converted to GA_12 _by KO and KAO, and in the third stage, GA_12 _and GA_53 _are converted to bioactive GAs by 2-oxoglutarate-dependent dioxygenases, GA20ox and GA3ox, whereas the conversion of bioactive GAs to inactive form is catalyzed by GA2ox [13,14]. Studies suggested that *GA20ox*, *GA3ox *and *GA2ox *encode enzymes important in regulation GA contents and homeostasis. Overexpression of *GA20ox *and *GA3ox *increased GA content and enhanced stem elongation [40-44], whereas overexpression of *GA2ox *reduced GA content and produced dwarf phenotype [17,37,43]. However, overexpression of genes in early steps of GA biosynthesis pathway caused no changes in plant morphology and levels of active GAs [45], suggesting that later steps were more rate-limiting in bioactive GAs biosynthesis. In this study, we demonstrated that *GA20ox *and *GA3ox *genes were up-regulated in a wheat hybrid, whereas *GA2ox *gene was down-regulated in hybrid. One of the genes in early steps, *KAO*, was also upregulated in hybrid, whereas *KS *gene was down-regulated in hybrid. Considering their roles in GA biosynthesis, upregulation of *KAO *in hybrid might produce more early intermediates for bioactive GAs, upregulation of *GA20ox *and *GA3ox *in hybrid, and down-regulation of *GA2ox *in hybrid could result in the higher GA contents in hybrid.

Several regulators of GA biosynthesis genes have been found in plant. *RSG *is a bZIP transcription factor that activates expression of *KAO *gene [46], and *14-3-3 *protein can repress expression of *RSG *by participating in GA induced feedback down-regulation of *RSG *[47,48]. In this study, we found that *RSG *was expressed as mid-parent value in hybrid, and *14-3-3 *was down-regulated in hybrid, which might provide a regulating mechanism to ensure the up-regulation of *KAO *genes in hybrid.

### Differential expression of genes involved in GA response pathways might be responsible for the increased internode elongation in hybrid

Studies indicated that genes in GA response pathway played crucial roles in GA-regulated developmental processes, and expression patterns of these genes were linked tightly to GAs biosynthesis and catabolism, and affected by GAs concentration [14]. *GID1 *is a soluble GAs receptor and overexpression of *GID1 *lead to elevated sensitivity to GAs signal and increased plant height [33]. In this study, we found that transcript level of the putative wheat homolog of *GID1*, *TaGID1*, was up-regulated in hybrid, suggesting that higher GAs contents might enhance expression of GAs receptor gene in hybrid, or the sensitivity to endogenous active GAs in hybrid may be higher than parents. In GA signal pathway, *GAMYB *is an important positive regulator which binds the target genes and activate their transcription [49]. In this study, the wheat homolog of *GAMYB*, *TaGAMYB*, was also up-regulated in hybrid. Thus, the enhanced transcription of *TaGAMYB *in hybrid might result from the increased GA level in hybrid or elevated sensitivity to GA signal. Moreover, upregulation of *TaGAMYB *in wheat hybrid could lead to the enhanced expression of *GAMYB *regulated target genes in hybrid and thus regulating hybrid vigor in growth and plant height. As a negative regulator in GA response pathway, *GAI *restrain the transcription of target genes in the GA signal pathway, the so-called repress-derepress hypothesis of GAs signal response [50]. Expression of *GAI *in hybrid was of mid-parent value, suggesting that in hybrid the negative regulation of *GAI *in GA response pathway was weaker than in higher parents, thus enhancing GA signal transduction.

Through specific response pathway, expression of target genes in GA signal pathway is induced, and expression of these target genes may directly promote cell division and cell elongation, and thus result in stem elongation. Up to date, known target genes of GA signal include expansin, *XET *(xyloglucan endotransglycosylase) and *GIP *(gibberellins induced protein). Semi-quantitative PCR analysis in our previous study revealed that transcripts of 4 β-expansin genes were up-regulated in wheat hybrid [39]. The function of expansin is unlocking the network of wall polysaccharides and permitting turgor-driven cell enlargement, and β-expansins may act selectively on cell walls of monocots, whereas α-expansins have been shown to loosen more effectively on cell walls of dicots [51,52]. Taken together, the higher transcription level of the 4 β-expansin genes in hybrid is likely to promote internode elongation in hybrid. Sharing high sequence similarity with *GAST *of tomato and *GASA *of Arabidopsis, *GIP *gene family in *Petunia *is induced by GAs and involved in stem elongation. Result in this study showed that transcript of wheat *GIP *homolog was up-regulated in hybrid. In *Petunia*, *GIP1 *and *GIP2 *mainly participated in cell elongation, whereas *GIP4 *and *GIP5 *were mainly involved in cell division [38]. As wheat homolog of *GIP, TaGIP*, is homologous to *Petunia GIP5*, we speculated that its higher transcription level in hybrid might promote activity of cell division in hybrid and thus contribute to hybrid vigor in stem growth. *XET *gene is also a GA-induced gene and participates in reconstruction of cell wall structure and promotes cell elongation. We found that transcript level of *XET *gene in wheat hybrid was close to the higher parent, which might also contribute to increased internode elongation in hybrid to some extent.

In order to verify whether GA response pathway genes were responsive to exogenous GA application, expression patterns of *GAMYB*, *GIP *and *XET *were determined before and after GA treatment. We found that transcript level of positive regulator *GAMYB *was up-regulated and negative regulator *GAI *was downregulated after applying exogenous GA_3_. Expression of *GIP*, a target gene of GA response pathway, was also enhanced after GA treatment. This result confirmed the conclusion that expression of GAs responsive components was linked to the GAs biosynthesis and affected by GAs concentration [14]. Considering that GAs content was higher in hybrid, at the same time GAs responsive genes were differentially expressed between hybrid and parents, we suggested that the higher GAs content in hybrid could be responsible for the differential expression of GAs responsive genes between hybrid and parents. Transcript level of *XET *gene was slightly decreased after GA_3 _treatment, which was unexpected. Studies suggested that *XET *was regulated by both GA and BR [36], and we found that transcript level of *XET *in wheat hybrid was close to the higher parent, thus the expression pattern of *XET *in hybrid might be explained by involvement and cooperation of different plant hormones, however, this will need further investigation. Since both GA and BR are related to the internode elongation in plants, further studies are also needed to investigate the how these two phytohormones are cooperatively regulating heterosis in plant height.

### A proposed model of GA and heterosis in wheat plant height

**Figure 2:**
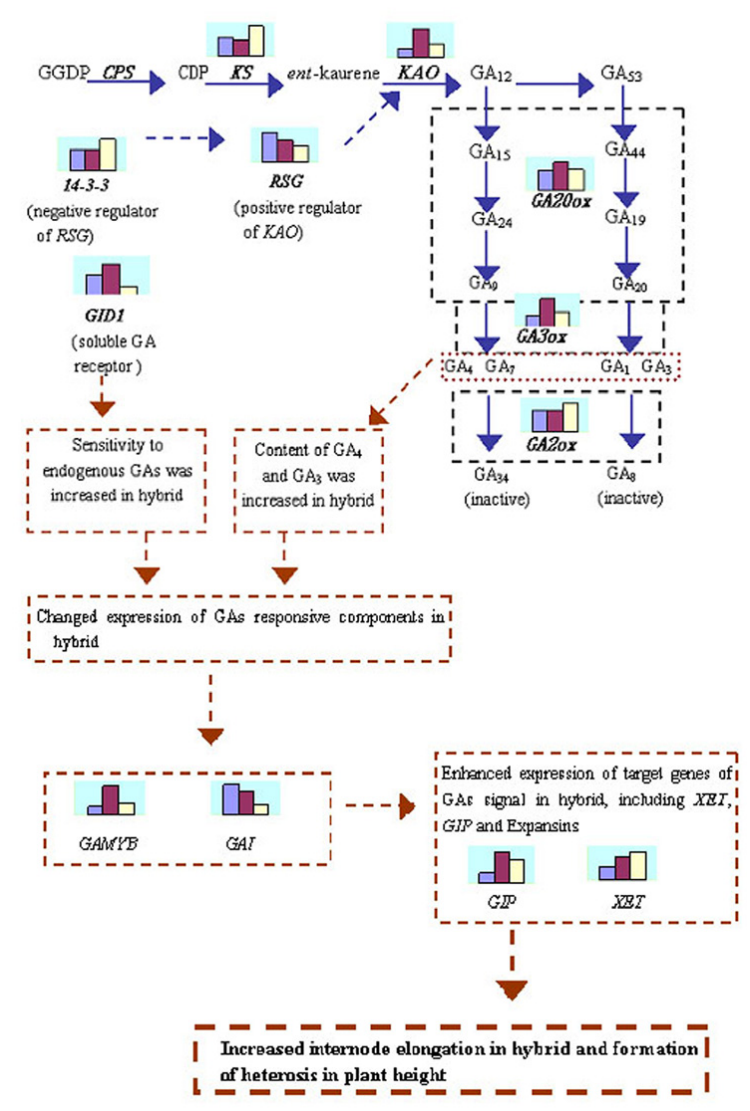
A proposed model for GA biosynthesis and response pathway in regulation of heterosis in plant height. Differential expressions of genes in GA metabolism and response pathways are listed in the box, with the bar heights representing the expression levels of female (left bar), hybrid (middle bar) and male (right bar) parent.

Taken the results above together, a model for GA biosynthesis and response pathway in regulation of heterosis in plant height of wheat was proposed (Fig. 2). In the first part of the model, combination of upregulation of genes enhancing bioactive GA production and down regulation of genes deactivating bioactive GA resulted in higher level of endogenous GAs in hybrid. In the second part of the model, upregulations of positive components, including GA receptor *GID1 *and *GAMYB *in GA signal transduction and response pathway, and downregulation of negative component of GA response pathway, *GAI*, could result in enhanced sensitivity to endogenous GAs signal in hybrid. And in the third part of the model, expression GA response target genes, including expansins, *GIPs *and *XET*, were up-regulated due to increased GA concentration and enhanced sensitivity to endogenous GAs in hybrid could promote cell division and cell elongation, and thus contributed to the increased internode elongation in hybrid and therefore heterosis in wheat plant height. It must be noted, however, that differences in mRNA quantity might not necessarily reflect the differences in the protein level, more works in protein level and enzymology are needed.

## Conclusion

Our results clearly shown that *GA20ox *and *GA3ox *genes were up-regulated in a wheat hybrid, whereas *GA2ox *gene was down-regulated in hybrid, which, in turn, could contribute to the increased bioactive GAs contents observed in hybrid. This increased GA contents could lead to the enhanced transcription of *GAMYB*, a positive regulator in GA signal pathway, in hybrid, which could then result in the increased expression of GA responsive target genes, including expansins, *XET *and *GIP*, and promoted elongation of internodes in hybrids. Therefore, this study provides molecular evidence for the heterosis in plant height in wheat and possibly other cereal crops.

## Methods

### Plant Materials and estimation of heterosis in plant height

Two spring wheat lines 309-1, AIM-11 and their F_1 _hybrid, two winter wheat lines, AI9, JiAi8 and their F_1 _hybrid were used for this study. For greenhouse experiment undertaken in 2005, the germinated seeds of each genotype were vernalized for 5 weeks at 0–4°C, 15 seeds of each genotype were planted in plastic pot (40 cm × 40 cm), which were placed in a greenhouse under 25°C/15°C (day/night) temperature. For field experiment in 2006/07, only the hybrid AI9/JiAi8 and its two parental lines were used, and the materials were planted with three replicates in October 5^th ^of 2006 in the field and plant height was recorded from 15 plants for each replicate in May 17^th ^of 2007. To determine the heterosis in plant height, final plant height and length of five internodes from the hybrids and their parents were determined, and paired t-test was used to determine the significance of differences between hybrids and their corresponding parents. The basal 1 cm-long portion of the uppermost internode (1 cm above the uppermost node) was excised in the greenhouse condition and stored at -80°C for GA content analysis and RT-PCR analysis from the hybrid Ai9/JiAi8 and its parents at the stage when the young ear was emerging from flag leaf and the uppermost internode was elongating rapidly. This portion of internode included the intercalary meristem (IM) zone and the elongation zone where the cells undergo continuous division and elongation.

### GA treatment

For GA treatment, the whole uppermost internode including ear of the genotype Ai9 was excised and soaked in 200 μg/L GA_3 _solution or distilled water (control). After 6 h of treatment, the basal 1 cm part of the internode was excised and stored at -80°C after frozen in liquid N_2 _for use.

### Measurement of endogenous GAs content

Gibberellins (GAs) content in the internode was determined as described previously [53,54]. Briefly, the internode tissues were ground into fine powder in the liquid nitrogen and 0.5 g samples were extracted in 80% methanol with 2 ng each of ^2^H-labeled GAs (2H-GA_3_, 2H-GA_4_) as internal standards. After a series of organic extractions, the extracts were purified through C^18 ^column, and then analyzed by gas chromatography-selected ion monitoring. Three independent samples were measured, and t-test was used to determine the significance of differences in GA content between hybrid and its parents.

### RNA isolation and Reverse-transcription

Total RNA was isolated and purified from each sample using TRIzol according to the manufacturer's instructions. Two microgram total RNA of each sample was used for first-strand cDNA synthesis in 100 μl reaction containing 20 μl 5 × RT buffer, 20 μl 2.5 mM dNTP, 10 μl 50 mM T15 anchor primer, 2.0 μl RNase Inhibitor (20 U/μl), 2.5 μl reverse transcriptase (50 U/μl). Reverse transcription was performed 42°C for 45 minutes with a final denaturation at 95°C for 5 minutes.

### cDNA cloning and primer design

For the genes that the sequences are not available in wheat, gene sequences from other plant species were used to search the wheat ESTs with high similarities in GenBank. The specific PCR primers (Table 6) were designed based on these wheat EST sequences and used to amplify cDNA. Amplified products were cloned and sequenced to verify the specificity of PCR primers. For those wheat genes, specific primers were designed and amplified products were also cloned and sequenced to verify identity of PCR products. Sequence analysis was performed using DNAMAN.

### Real-time quantitative PCR

A 300 or 150 bp β-actin gene fragment was amplified as an endogenous control using the primer pairs in Table 6. For real-time quantitative PCR, cDNAs from three biological samples were used for analysis and all the reactions were run in triplicate and included no template and no reverse transcription controls. Quantification results were expressed in terms of the cycle threshold (CT) value determined according to the manually adjusted baseline. Relative gene expressions in hybrid and parents were determined using the method as described previously [55,56]. Briefly, differences between the CT values of target gene and β-actin were calculated as ΔCT = CT ^target ^– CT^β-actin^, and expression levels of target genes relative to β-actin were determined as 2 ^-ΔCT^. For each sample, PCR was repeated three times, and the average values of 2 ^-ΔCT ^were used to determine difference in expression between hybrid and parents.

### Histological analysis

Internode tissues of about 1 cm above the uppermost node, which containing IM (intercalary meristem) and elongation zone, were fixed in solution of formalin: acetic acid (FAA):70% ethanol (1:1:18). For hematoxylin staining, plant materials fixed in FAA were dehydrated through a graded ethanol series and embedded in Technovit 7100 resin. Microtome sections (3–5 μm thick) were stained with hematoxylin, and were observed and photographed using microscope.

## Authors' contributions

Y. Z and Z. N carried out the molecular analysis, Y.Y and X. N participated in the GA content measurements, Q. S designed that experiment and helped with the writing of the manuscript. All authors read and approved the final manuscript.

**Table 6:** PCR Primer sequences, length of PCR products and similarity with known genes

**Genes**	**primer sequences**	**length of PCR product (bp)**	**Similarities with homologous genes**
*Actin*	S 5'-TCATTGGTATGGAAGCTGCTGAATC-3' A 5'-CCTGACTCATCATACTCGCCCTTCG-3'	300	*Oryza sativa *actin mRNA, complete cds Identities = 279/330 (93%)
*Actin*	S 5'-GGAAGTACAGTGTCTGGATTGGAGGGT-3' A 5'-TTCAGAAGACCCAGACAACTCGCAAC-3'	150	*Oryza sativa *actin mRNA, complete cds Identities = 140/150 (93%)
*14-3-3*	S 5'- CACTATGTCTGGGGTCG -3' A 5'- ATTTAGGACTTGCTGGCA -3'	340	*Triticum aestivum *14-3-3 protein Identities = 100%
*RSG*	S 5'-TGGGCTACCGGACTACGCCAAG-3' A 5'-CCT TGGAACTTGACCTGCCGCTT-3'	367	*Nicotiana tabacum *mRNA for bZIP transcriptional activator RSG Identities = 67%
*KAO*	S 5'-ACAACTGCCTGGCCAAGATCACCAG-3' A 5'-GTGACAACTTTGACTCATCCGCGACAACAA-3	184	*Hordeum vulgare KAO *mRNA Identities = 166/184(90%)
*KS*	S 5' CCCCTGAACTTTCTGATGCTTGCATAT-3' A 5'-TTGAAGGACTGTACTTCTCAACCAATGCT-3'	137	*Hordeum vulgare *KS-like gene Identities = 116/127 (91%)
*TaGA2ox-1*	S 5'-TCGCTCCGCCTAAGCCACAG-3' A 5'-CTGCAACTCAAGCAGTCATCCCTC-3'	104	*Oryza sativa *mRNA for *OsGA2ox1* Identities = 73/104 (71%)
*TaGA20ox-2*	S 5'-GCTGAGCCAGGGCGTGGAGAAG-3' A 5'-CCATGAAGGTGTCGCCGATGTTG-3'	260	*Oryza sativa *gibberellin 20-oxidase 2 (Sd-1) gene Identities = 240/260 (92%)
*TaGA20ox1D*	S 5'-AGCACTACCGGGCGGACATGAA-3' A 5'-GCCATCCATCCATGCTTCTTCGTAC-3	260	Wheat GA20-oxidase 1 gene homoeologous *TaGA20ox1D* Identities = 100%
*TaGA3ox2-1*	S 5'-GTACATGGGCGTGCGCAAGAA G-3' A 5'-GCACGCATCCACCAGCATCATC-3'	219	Wheat GA3-oxidase 2 gene homoeologous *TaGA3ox2-1* Identities = 100%
*TaGA3ox2-2*	S 5'-GTACATGGGCGTGCGCAAGAAG-3' A 5'-CAGCTAAGCTACCAGCCCACCATG-3'	258	Wheat GA3-oxidase 2 gene homoeologous *TaGA3ox2-2* Identities = 100%
*TaGA3ox2-3*	S 5'-GTACATGGGCGTGCGCAAGAAG-3' A 5'-GCTAATCTAACAGCCCGCCACCAT-3'	260	Wheat GA3-oxidase 2 gene homoeologous *TsGA3ox2-3* Identities = 100%
*GAI*	S 5'-GCACATTCCTGGACCGCTTCACC-3' A 5'-GCAGCCTTCCTTCTCCTCCACCTTG-3'	422	*Triticum aestivum Rht-1 *gene Identities = 100%
*TaGID1*	S 5'-CCACCATCGGCTTCTACCTGCTGTC-3' A 5'-GGCGAGCTCATCCACGACGAGAC-3'	133	*Oryza sativa *mRNA for *GID1* Identities = 120/133 (90%)
*GAMYB*	S 5'-CGTGAGAAGTTCAAGTTCCTCTGT-3' A 5'-AAGTTTTCAGGATGAGACGAAGTG-3'	287	*Triticum aestivum MYB3 *gene Identities = 100%
*GIP*	S 5'-GGGACGCAGTACAAGAAGG-3' A 5'-GGAAACTGGGAGGGCAAT-3'	300	*Petunia hybrida *mRNA for (gip5 gene) Identities = 64/69 (92%)
*XET*	S 5'-GCCCTTCGTCGCCTCCTAC-3' A 5'-CGGCACAACAACAACTAGTGGTAG-3'	300	Wheat mRNA for endo- xyloglucan transferase Identities = 100%
